# Mechanisms of Participation in Vocational Education and Training in Europe

**DOI:** 10.3389/fpsyg.2022.842307

**Published:** 2022-05-30

**Authors:** Paul Milmeister, Merlin Rastoder, Claude Houssemand

**Affiliations:** Institute for Lifelong Learning and Guidance, Department of Education and Social Work, University of Luxembourg, Esch-sur-Alzette, Luxembourg

**Keywords:** VET, Europe, vocational choice, educational policy, labor culture

## Abstract

This article aims to analyze vocational education and training in Europe and to model mechanisms of educational and vocational choice. First, we expose the differences between VET approaches in Europe. Secondly, a sociological analysis is provided. When VET systems were first created, aspects such as work culture or diverging political concerns led to different responses in the various countries. Thirdly, we present a psychological approach of the educational and vocational choice which draws on a process where profession images are compared with one’s own self-image. Finally, we present an integrated explanatory model of the vocational choice, based on sociological and psychological dimensions. In conclusion, we propose several plans of action in order to support and inform students regarding educational choice and to improve valorization of the VET track.

## Introduction

Since the early 2000s, the EU has a clear public policy of developing the knowledge society. Education and training would be the two essential drivers of this aspiration, with them conducive to economic growth, sustainable development, research and development, innovation, productivity and competitiveness. This training perspective was initiated by the Copenhagen process in 2002 (started in Bruges in 2001), confirmed in Riga in 2015, with its targets to be achieved by 2020. To do this, programs such as “A new start for Europe: My agenda for Jobs, Growth, Fairness and Democratic Change” are implementing actions to promote vocational education and training (VET) in Europe, such as promoting vocational training in all its forms, developing quality assurances in VET, improving access to VET and qualifications for all, strengthening key skills in VET programs and allowing systematic access to initial and ongoing vocational training programs for VET teachers, trainers and mentors (Journal officiel de l’Union européenne, 2015).

Nevertheless, despite this common European desire to develop vocational training and education, differences appear between member states and/or associated states. The importance of this method of professional specialization is not the same across countries and cultures. It therefore seems useful to question the empirical reality of European VET policies and to try to pin down the reasons for the variability of the way it is implemented and the results.

This article presents a brief overview of participation in vocational education and training in Europe, followed by explanations of the observed differences, taking into account sociological and psychological aspects. Ultimately, this work is intended to study the mechanisms of participation in vocational education and training in Europe. It also seeks to open avenues of reflection and action on the elements that impact young people’s decisions to follow VET and apprenticeships. This should enable better understanding and management of the adequacy between youth training and market demands.

## Vocational Education and Training Across Europe

### Participation in Vocational Education and Training and Work-Based Learning

**FIGURE 1:**
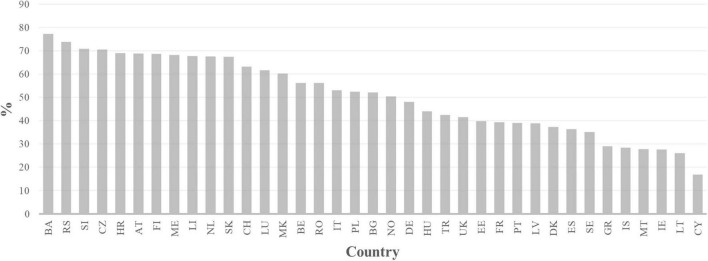
Students in upper secondary VET in% (2019) Note. Data source: Eurostat (2021).

**FIGURE 2:**
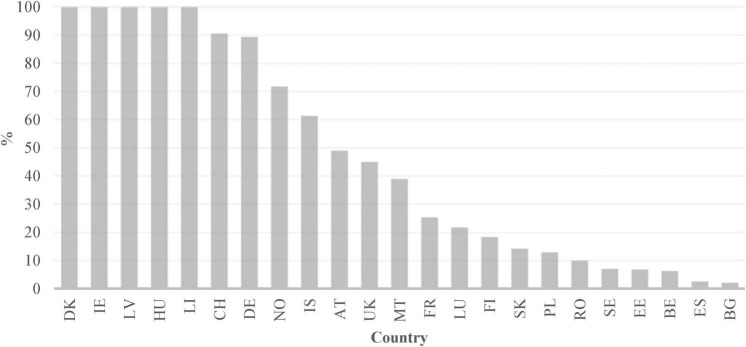
Students in a work-based training among those in VET in% (2019) Note. Data source: Eurostat (2021). Data is missing for Czechia, Cyprus, Croatia, Greece, Italy, Netherlands, Portugal, Lithuania, Slovenia, Montenegro, North Macedonia, Serbia, Turkey and Bosnia and Herzegovina.

When we take a look at VET across Europe, we see that it is a highly heterogeneous educational sector with different approaches to it. The percentage of students in VET in secondary education varies greatly from one European country to another (see Figure 1). On the one hand, countries such as Bosnia, Serbia, Slovenia, Czechia, Croatia, Austria, Finland, the Netherlands, Slovakia or Switzerland have participation rates above 60%, while Cyprus, Lithuania, Ireland, Montenegro, Iceland or Greece are below 30%. Another remarkable difference between countries is the proportion of students in work-based learning (see Figure 2). In Denmark, Hungary, Ireland, Latvia, Switzerland and Germany, more than 80% of VET students are following a work-based training (i.e., in companies), while in other countries, such as Sweden, Estonia, Belgium, Spain, or Bulgaria, less than 10% of VET students are being trained this way (Eurostat, 2021). The landscape of vocational education and training in Europe is thus one with a quite complex pattern. The following sections will address a series of further elements which help to deepen our view on VET’s reality throughout Europe.

### Classifications of Countries According to Vocational Education and Training Characteristics

A first step in understanding differences in VET is to attempt to classify the observed systems. Drawing on a typology by Hanushek et al. (2011), CEDEFOP (2018) tries to do this and operates a classification of European countries into six types. The distinction is based on the two criteria we have just presented: participation in VET and the proportion of students undergoing work-based learning. In short, this typology ranks countries on a scale ranging from a situation where many students are in VET and also in work-based practical training, to a situation where few students are in VET, and vocational training is mainly school-based. Type 1: Countries practicing the dual system, with a high percentage of students in VET and work-based learning (Germany, Switzerland, Denmark). Type 2: Countries with a similar distribution between a purely school-based education and a dual system (Netherlands, Austria). Type 3: A country with few students in VET, but with a high proportion of work-based training (Hungary). Type 4: Countries with an average percentage of students in VET and work-based learning (Great Britain). Type 5: Countries with education based mainly in school, with a separate apprenticeship system and few students in work-based training (Belgium, Bulgaria, Czechia, Croatia, Italy, Luxembourg, Malta, Poland, Romania, Slovenia, Slovakia, Finland). Type 6: Countries with a school-based education system with limited vocational training taking place at school (Greece, Spain, Cyprus, Portugal, France, Estonia, Latvia, Lithuania, Sweden).

It is no exaggeration to say that VET in Type 1 countries is an entirely different affair than in Type 6 countries. Type 1 appears to be a relatively homogeneous group of countries culturally and geographically close to Germany. Type 3 consists of a single country, Hungary, but can be likened to type 1, given the high percentage of students in work-based training. Type 6 is strongly varied. On the one hand there is a group of countries bordering the Mediterranean (France, Spain, Greece) and on the other, countries of the Baltic region (Sweden, Estonia, Lithuania). Type 5 has an even greater diversity, comprising Nordic, Southern, Western and Eastern countries.

### Public Image and Vocational Education and Training Information

Vocational education and training, and education in general, are not taking place in a neutral surrounding. The national contexts mentioned above differ in terms of the social conditions in which the school systems are embedded. The general public image of VET within society can be a good measure of acceptance of VET in a given country and an explanatory element of participation in VET across Europe.

Vocational education and training is sometimes seen as an effective way to increase employment prospects for young people who do not have the resources, motivation or skills to pursue further education (Shavit and Müller, 2000; Eichhorst et al., 2015). Polls show that vocational education is often perceived as a solution for academically low performers. Three out of four European citizens (75%) agree that students with low grades are oriented toward vocational education in their country, and 63% agree that it is easier to obtain a qualification in vocational education than in general education streams (CEDEFOP, 2017). The public perception of vocational training is sometimes rather negative, not least because for some, it is a dead end and a second-choice education (Eichhorst et al., 2015).

Across Europe, VET appears to be more entrenched and valued in some countries than in others. According to Mulder (2012), in countries with a rather Nordic outlook, VET has a good reputation, while in more southern oriented countries, VET is significantly less valued than general secondary education. CEDEFOP (2017) thoroughly studied the perception of VET by European citizens. At first sight, VET seems indeed to have some image problems since the majority of Europeans agree that general secondary education has a better image than vocational education (yes 72.7%; no 17.3%; don’t know 10%). In truth, this is only relative, since about two thirds of Europeans (68%) also believe that vocational education has a positive image in their country. The countries with the most respondents saying that vocational training has a positive image are Malta, Finland, Czechia, the United Kingdom and Italy (between 75 and 89%). Respondents saying that it has a negative image are the most numerous in France, Hungary, Belgium and the Netherlands (between 41 and 44%). These numbers show a fairly pronounced range of opinion and refute the idea that the appreciation of VET simply follows a north/south logic. When we put the perception of VET in relation to the Cedefop typology presented above, we can see that these two do not follow a common logic either. Of those countries favoring general education (Type 6) almost all have an average, but not negative appreciation of VET, with the exception of France. Further, one would expect that the countries with a “dual system” (Type 1) would be those with the most positive view of VET, but in Germany “only” 71% have a positive opinion of VET, and this is 60% in Denmark. Interestingly, VET seems to be appreciated by its graduates to a greater extent than general secondary education is appreciated by its graduates. Many respondents who have taken vocational education themselves say they would recommend it (60%) to a young person about to enter higher secondary education, rather than general education (15%)^1^. For those who received general secondary education, only 38% said they would recommend it, compared to 26% who would recommend vocational education (CEDEFOP, 2017).

Many people in eastern European countries recommend vocational education, while respondents in Ireland, Luxembourg and Sweden are the most likely to recommend general secondary education. Germany and Britain are among the countries where VET is not likely to be recommended a lot, but where many people say that it depends on the individual student. These findings allow many interpretations. Countries where VET is highly recommended are in many cases formerly socialist states. One obvious explanation would then be a traditional value for practical and professional training. At the same time, some of these countries’ economies are heavily focused on the production of goods rather than on services, which fosters opportunities in crafts and industry. Conversely, countries where respondents value general secondary education are often countries where employment in the tertiary sector is important.

**FIGURE 3:**
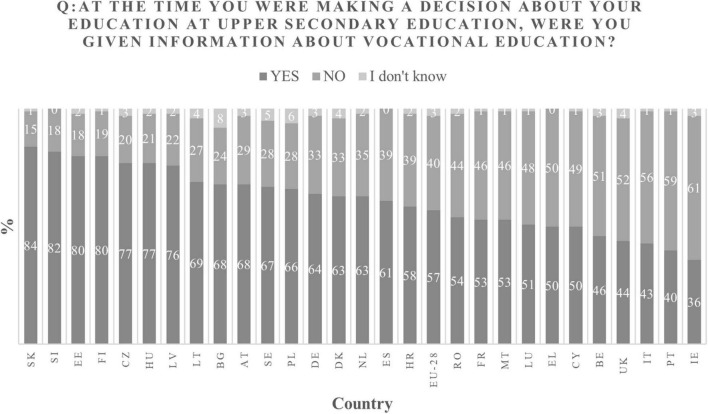
Information on VET given to students by country. Note. Adapted from CEDEFOP (2017, p.26).

Similarly as VET systems do not exist in a neutral surrounding, the European students do not all live in similar contexts either. This is obvious for instance regarding the information that is provided to students (see Figure 3). The countries where the most students say they received information on VET before making a decision are Slovakia, Slovenia, Estonia and Finland, while those with the fewest students having received this type of information are Ireland, Portugal, Italy and the United Kingdom. It appears that the level of information given on VET is fairly strongly correlated (Pearson index of .669) with the participation rate in VET at the national level (CEDEFOP, 2017). It is therefore plausible to assume that there is a causal link between the provision of information on VET and the decision to choose this educational route.

### Transition to the Labor Market

A major contextual aspect of school systems, which obviously affects the students within them in their choices, is the surrounding economic situation and the employment outlook. After the 2008 financial crisis, many European countries experienced high rates of youth unemployment. It then emerged that countries applying the dual system (Austria, Germany, Switzerland, Denmark) were less affected by this problem, which led to the assumption that there is a link between the two. More concretely, it was assumed that the training of young people in companies allows a secure transition into employment, due to the tendency of training companies to hire their apprentices (Eichhorst et al., 2015; Kleinert et al., 2018). According to Eichhorst, “(.) the dual system, which is most prominent in a number of continental European countries, is more effective than alternative academic or training education at helping youth transition into employment” (2015, p.331). However, Mulder (2012) makes the opposite reasoning, and asserts that labor market conditions determine students’ training choices. Mulder’s argument is that in a situation of economic crisis and high unemployment, greater flexibility is required on the part of young people, and that vocational training of too specific a nature can then be seen as a choice that reduces prospects, as opposed to more general training.

Some studies suggest that there is a positive effect of VET on employability. For young adults, the employment rate of those with vocational training in secondary school is higher than that of those with general secondary education, regardless of the national context (OECD, 2017; CEDEFOP, 2018). However, the results also show that, in terms of the quality of employment, the advantage of VET over general education depends on the national context. Countries with a dual system and those offering both school and work-based learning offer better chances of escaping low-skilled employment. While VET reduces the risk of unemployment compared to general-type education in countries with predominant general secondary education, it increases this risk in countries with a school-only VET system. Thus, unemployment protection and reduced likelihood of low-level work through VET (so-called safety net effect) is observed only in countries with the dual system (Type 1) and in those with a school-based and work-based VET (Type 2) (CEDEFOP, 2018). These findings are echoed by the work of Shavit and Müller (2000), which have found a safety net effect of VET particularly in the dual system countries.

**FIGURE 4:**
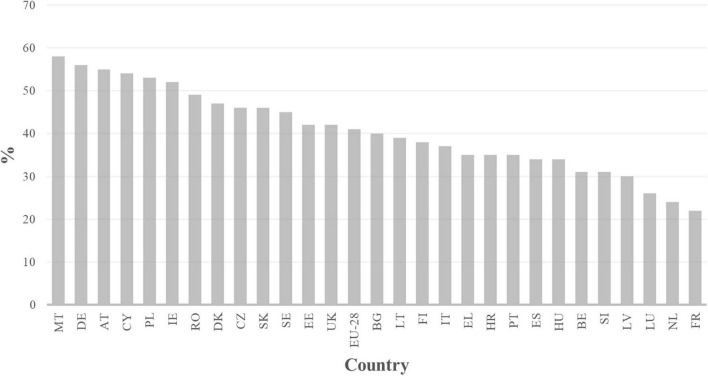
Index of VET image in relation to the labor market (percentage of agreement). Note. Adapted from CEDEFOP (2017, p.45). This index is created with the degree of agreement with 4 items: ‘People in vocational training acquire the skills that employers in [country] need; Vocational training allows you to find a job quickly after obtaining a qualification or diploma; Vocational education leads to high-paying jobs; vocational training leads to highly valued jobs in [country].’

In its opinion poll on VET, Cedefop asked four questions related to the usefulness of VET training as a preparation for the labor market. They deal with the usefulness of the skills learned, the chance to find work quickly, the level of pay to be expected, and the image of the expected positions. Figure 4 resumes the level of agreement with the four questions. It shows that there are meaningful differences between countries like Malta, Germany and Austria on the one hand and France, the Netherlands and Luxembourg on the other. While the former seem to offer interesting opportunities for young people coming out of VET, this does not seem to be the case in the latter. The United Kingdom takes an intermediate position. France’s low score seems to be mainly related to the poor pay of prospective jobs, whereas in the Netherlands it would be rather because of their poor public image (CEDEFOP, 2017).

## Sociological Aspects Related to Vocational Education and Training

In our effort to better understand the differences in participation in VET throughout Europe, it is essential to highlight the contextual aspects in which the educational systems and the students are embedded. The following sections present some historical and sociological explanations for the reality of VET that we observe today.

### The Historical Development of Vocational Education and Training in Europe

In order to make sense of the high variability of VET and the six types of countries regarding it, it is necessary to make a short excursion into the historical developments that led to the implementation of professional training. Every European’s educational choices are influenced up to this day by the historical underpinnings of the context in which he or she evolves. In Europe, vocational training has emerged in different forms in different countries. Greinert (2005) has developed a typology featuring three “classic” models of VET in Europe, with each being specific responses to the first industrial revolution and the erosion of traditional models of craft training. According to him, the basis for this differentiation was provided by different national structures and cultures, which he summarizes as “labor cultures”.

Bercusson et al. (1992) as cited by Greinert (2005) describe three paradigmatic European contexts with regard to labor law. These are in Britain, France, and Germany. They consider that the concerns reflected in national legal frameworks are not the same. According to Bercusson et al. (1992), these differences are played out in parallel on a security – freedom opposition. The right to social security was introduced relatively early in Germany, with individual freedom being little regarded. In France, much weight has been given to political freedom (political expression, action and organization) in relation to social security. In the United Kingdom, freedom has also prevailed over security, but rather in the form of protecting market freedom of action. For Bercusson et al. (1992), freedom is understood in France as being guaranteed by the state, and not, as in Britain, as a liberation from the state. This analysis can be summed up as follows: Great Britain and France have chosen to value the freedom of the individual at the expense of the collective (freedom of the citizen and political actor on the French side, freedom for the entrepreneur and economic actor on the British side). Germany, for its part, has valued collective responsibility at the expense of guarantees for the freedom of the individual.

By applying the idea of labor cultures to the organization of VET, Greinert (2005) argues that Britain has set up a model based on liberal economy (market model), France a model based on state bureaucracy, translating in practice into a school-based model and Germany a dualistic corporatist model where state and companies meet and resulting in the dual system of apprenticeship. In this view, the British market model is a model that trusts the regulating power of the markets to ensure the provision of VET. The state has very little involvement in the operation of the system. The characteristics of the market-based model are: (a) the quantitative relationship between training needs and available coverage is market-regulated (b) the nature of professional skills is determined by their usefulness in the labor market; (c) there are few standards for training. They can be purely school-based, very practical or alternating between school and business. There are few generally accepted certificates; (d) the costs of vocational training are borne by learners or by companies; (e) in countries applying the market-based model, an important distinction is made between general education and specific VET. The first takes place in state schools and the second on the basis of an agreement between market players.

The French school-based model trusts the state to organize and regulate VET. For Greinert (2005), this model is as follows: (a) the quantitative relationship between training needs and available supply is established by public bodies; (b) the nature of professional skills depends less on their immediate application in the workplace; (c) the school-based model is strongly differentiated between courses; (d) school-based VET is publicly funded. Limits on public funds can compromise the access of some cohorts to the existing limited supply. As a result, school-based VET would tend to be “elitist” and specialize in more advanced professional skills; (e) in the medium term training courses tend to climb the qualification ladder, and new training needs to be established for people with lower academic ability. The result is problems satisfying mass VET needs.

The German model or dual system trusts the social responsibility of the actors involved to organize and regulate VET. The state is involved in the VET system in a regulatory manner, but is far from being able to act alone. The dual system is the result of consultation and collaboration between the state, employers and wage earners. Greinert (2005) presents it as follows: (a) the dual vocational training system forms an area of training largely isolated from general education, with its own organizational structure and legislative basis; (b) the company is the key place to learn in this cooperative system, and in-company training (apprenticeship) is the standard; (c) the nature of the training is determined by the employer or organizations representing the interests of certain groups of employers. Educational profiles are established through an agreement between employers, trade unions and public bodies, and are enshrined in national legislation; (d) apprenticeship costs are generally borne by businesses, but can be deducted from taxes. Apprentices receive monetary compensation from their employer. The operating costs of the vocational school are covered by public funds; (e) the dual system is derived from a traditional context of crafts, two elements of which have been preserved: the principle of work-based learning, and the principle of self-regulation.

For Greinert (2005), the other VET models that exist in Europe are either variations or combinations of the three models cited. These major models of thought and organization (France, Germany, Great Britain) can therefore be identified as the base of the observed diversity. The different organization of these models means that neither access to VET nor its internal way of functioning is comparable from one country to another. While in-company training plays only a secondary or even marginal role in some countries, it is the standard model in dual-system countries, which explains the high participation rates in this type of training in these countries.

Let us compare this typology with the six types classification of CEDEFOP (2018) to see how other European countries are placed. It appears that Britain and its market model seem to be an exception in Europe. Arguably the closest country culturally, the Netherlands, has a much stronger school-based system, with few students in work-based learning. The German model, with its traditional dual system and significant social consultation, is distinguished by very high rates of students following in-company training, and it turns out that this concerns only a few countries in Central and Northern Europe. In view of the simple participation figures in VET and work-based learning, Britain is close to the countries of the dual system, but the operation of the model is quite different. The French model finally, based on school and a highly valued general secondary education, seems to be the most widespread in Europe. Two groups of countries are close to it: Type 6, in which France itself is classified, and which is present mainly in the Mediterranean and the Baltic regions; and Type 5, which is present all over Europe. The latter is a school-based model, but with more students in VET. The largest number of European countries can therefore be classified as being close to the French “state” VET model.

### Social Organization and Vocational Education and Training

The structural and economic context in which an educational system is embedded can take an important role in an individual’s educational and vocational choice because it transcends the educational system’s logics and aims. The student has no choice but to think and act within the contextual logic that is forced upon him. In this sense, regarding organizational aspects, Maurice et al. (1986) for instance distinguish two main types of VET systems: “qualification spaces” and “organization spaces.” The base of this opposition is a comparison between France and Germany. The German system is described as a qualification space in which skilled workers and craftspeople have clearly defined professional identities. The advantages of this system are the standardization of qualifications, the resultant ease of mobility between companies for skilled workers, and the ability of well-trained young people to make a significant positive contribution to the company from the start. A disadvantage is the reduced inter-occupational mobility, as very specific training courses lock apprentices into specific activities. The French system, on the other hand, is described as an organizational space. VET in that country is rather school-based in nature and traditionally targeted at students who do not perform well at school. Advantages of this system are that it does not lock students into a restrictive vocational track, but offers more general training while gateways to other training and activities remain open. The disadvantages are that young people who leave VET are less well trained in practice, and companies have to conduct introductory programs for the young people recruited at their own cost. This investment incentivizes companies to retain workers once they have been trained. These differences end up playing a role in young people’s decision to engage or not in VET or in an in-company training.

### Social Inequalities and Educational Choices

Social stratification is a major reality in the educational field and it can have an important impact on student’s educational career and the decisions that they take. Since the post-war years, sociology has demonstrated the reproduction of social inequalities through school systems, which, far from keeping their promises of equal opportunities, sometimes contribute to cement established social structures (Bourdieu and Passeron, 1964, 1970; Boudon, 1973; Bowles and Gintis, 1976). Despite political awareness of the phenomenon and efforts to counter it, this phenomenon has proven to be persistent, albeit more so in some countries than in others. PISA^2^ studies show that social inequality remains an important factor in educational achievement: “Long-standing research finds that the most reliable predictor of a child’s future success at school – and, in many cases, of access to well-paid and high-status occupations – is his or her family. Children from low-income and low-educated families usually face many barriers to learning.” (OECD, 2019, p.50). In France, the National Centre for the Study of School Systems (CNESCO) considers that although French schools segregate students less blatantly than they did 50 years ago, they nevertheless tend to maintain many social and migratory inequalities within them, which are more hidden, less observable, but nonetheless very much present (CNESCO, 2016).

Social selection is quite visible within educational systems and the choice for VET tracks in particular can be seen as a social marker, or even as a mechanism to divert some students from higher educational tracks and more prestigious jobs. Actually, VET disproportionately attracts students from low-income backgrounds, not least because VET allows them to follow shorter educational pathways. Young people’s decision to follow VET options is strongly influenced by the opinion of their surrounding, and families often decide with a logic of social class maintenance. Children from more advantaged backgrounds need to study more extensively to maintain their status, while children from poorer backgrounds can maintain their status more easily. The costs of higher education are often a great burden for families of low socio-economic status. Compounding the problem, families from lower social classes tend to overestimate their children’s risk of failure, and also favor the educational pathways they have followed themselves (Di Stasio, 2017). Shavit and Müller (2000) have a bilateral approach and say that VET can be seen from two angles. On the one hand they note that it can be a way to increase the qualification and recruitment chances of those who follow it, and on the other hand, it can be seen as a mechanism of selection and social reproduction, allowing a part of young people to be put on a less prestigious educational pathway, a process known as “tracking.” Students from a disadvantaged socio-educational background are more often placed in less prestigious educational tracks, which reduces their chances and ambitions to pursue higher education. Consequently, their likelihood of occupying prestigious positions later on is also reduced. When students in VET take significantly less desirable jobs than those in other educational tracks, Shavit and Müller speak of a “diversion” effect. However, when students in VET have lower unemployment rates and lower employment rates in unskilled work than students in educational tracks other than VET at a comparable level, they speak of a “safety net” effect. The work of Shavit and Müller (2000) confirms that VET can reduce the probability of becoming unemployed, as well as the probability of being employed in unskilled work. Importantly, they also show that contrary to the often-assumed effect of mutual exclusion, VET can fulfill both a diversion and a safety net function. Based on Eurobarometer data, Di Stasio (2017) found that in countries with systems that can be categorized as “qualification spaces”, VET is seen both as a disincentive to undertaking further studies and as a safety net.

The sociological arguments presented in this section have shown the importance of the context to explain how differences in VET participation and organization emerge on the European level, and how the students are impacted by them on their individual level. The next part will focus on the individual mental aspects guiding the students in their decisions.

## Psychological Aspects of Professional Choice

### Building a Professional Identity

Alongside sociological and organizational aspects, young people’s educational choice is above all an individual process guided by psychological mechanisms. Research has shown that the level of planning for the future increases with age (Nurmi, 1991). The development of future orientations is not only related to individuals’ development of cognitive abilities, but also strongly linked to the motivational system, for which future representations of the self are important. In adolescence, social pressure regarding future orientation comes from the different life environments experienced by young people; mainly the family and school (Klineberg, 1967). Adolescents are pushed, explicitly or not, to define and commit to personal career plans. The development of future orientation can be influenced by social conditions, specifically by positive relationships with adults (Aronowitz, 2005; Kerpelman et al., 2008), but also discouraged by negative life conditions such as violence and poverty (McGee, 1984). Fusco et al. (2019) consider that the construction of a professional identity and professional self-definition is a major task in adolescence. It is initially based on childhood images of occupations that are stereotyped and idealized. These images are further anchored by more concrete experiences in the adult world. Occupational experiences and exploration of career possibilities during adolescence increase knowledge acquisition (Fusco et al., 2019).

The influence of peers and family on occupational identity is considered a relevant, albeit indirect, factor for the development of occupational identity (Skorikov and Vondracek, 2007). Liechti (2012) cites numerous studies in sociology, psychology and educational sciences that show that the family environment has a strong influence on the decision and choice of educational orientation. In the context of a traditional society such as Pakistan, Humayon et al. (2018) were able to show that this influence becomes preponderant. Among the three factors of self-interest, family influence and economic considerations, career choice is most strongly influenced by the family, and least of all by self-interest. The development of professional identity can be affected by changes in society, such as longer periods of education, more difficult access to the labor market for young people, postponement of the start of a stable married life or of marriage. All of which extend the phase of identity construction (Crocetti et al., 2012; Fusco et al., 2019).

### The Influence of Profession Images on Occupational Choice

Educational and vocational choices, and hence the choice for or against the vocational education and training track, seem to rely a lot on mental images. Piaget and Inhelder (1966) stated that throughout an individual’s life, his or her experiences create a multitude of mental representations, which then build up new images in the mind, or modify and enrich already existing images. For Gavard-Perret and Helme-Guizon (2003) the sensory experience generated by an object is provoked by external stimuli such as images, speech, sound, etc., and internal stimuli such as imagination and thought. The sensory information generated by these stimuli is integrated into the individual’s memory and produces images that can be recalled without the presence of the original object. This already existing image can be transformed or changed when the individual is exposed to different additional stimuli.

Brillet and Gavoille (2016) attempted to determine how representations guide individual occupational choices. They define the concept of mental image as a psychic construction of an absent object, enriched by social experience and influenced by the social representations of the group to which one belongs. The individual is exposed to various stimuli that constitute a perceptual experience of the profession, to which he or she adds personal reflections. All of these antecedents lead to representations of the profession, which are summarized under the concept of “profession image.” It is defined as a global representation of the job in the minds of individuals and corresponds to the set of mental representations formed following an individual’s exposure to different internal and external stimuli (Brillet and Gavoille, 2016). This profession image is at the center of the vocational choice, which is made by matching profession image and self-image.

The self-image of a person corresponds to the representation and evaluation that the individual makes of himself at different stages of his development and in different situations in which he finds himself (Doron and Parot, 1991). Early research related to vocational choice focused on the concept of “personality”. Holland (1966) created a theory of vocational choice which identifies interests that characterize different occupational profiles, but also explicitly recognizes the personality profiles of individuals who move toward a particular occupational/professional activity. He thus proposed six categories of vocational interests in his theory: “Realistic,” “Investigative,” “Artistic,” “Social,” “Enterprising,” and “Conventional,” each one corresponding to different personality profiles. While the concept of “self-image” as described by Doron and Parot (1991) and Brillet and Gavoille (2016) certainly echoes this typology, its focus lies on self-awareness and self-perception, and englobes thus the whole personality. This may also include gender or social class stereotypes.

When an individual finds elements in a profession image that are consistent with his or her self-image, this enables him or her to establish his or her own professional identity (Brillet and Gavoille, 2016). Brillet and Gavoille emphasize the fact that the image is the product of a perception and mental coding, but that part of the object is imagined by the individual. This means that the image always has a subjective side, and that it therefore presents a variability in the representation of the same object from one individual to another. It can be incomplete and far from reality. Simon (1957) conceptualized the idea of “bounded rationality”: an individual placed in a situation of choice can only take into account a limited amount of information to make a decision. Thus, for a young person lacking information, the profession image can be formed without direct exposure to the occupation, but only through speech and can therefore be an image of anticipation. Brillet and Gavoille consider that this is often the case.

**FIGURE 5:**
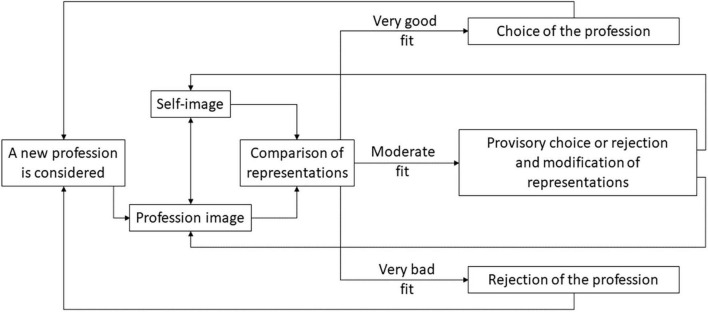
The selection of preferences for trainings or professional activities. Note. Reproduced and translated from Brillet and Gavoille (2016, p.59).

Based on Huteau (1982, 1992), Brillet and Gavoille (2016) use the cognitivist approach in counseling psychology to analyze how occupational choice is made. In this perspective, the individual compares his or her perception of an occupation with his or her self-image to determine the choice of occupation (see Figure 5). In other words, the occupational choice is a confrontation between the representations of professions, and mental representations of the self.

## Toward an Integrated Model of Vocational Choice

On the basis of the various elements presented above, we synthesize an integrated model of young people’s educational and vocational choice, and more precisely of the choice between vocational or general education in the European context. We have seen that VET is very diverse throughout Europe. Not only does participation in this type of education vary greatly, but the importance of work-based vocational education and training also varies substantially. A short review of aspects which are related to VET shows that there is no simple explanation for the diversity of VET systems and participation in them. Rather, it appears that a multitude of factors can be used to classify and analyze them.

We saw that the public image of VET does not necessarily turn out to be excessively positive in countries with high VET participation, and also does not seem to follow a straightforward geographical or cultural logic as some authors suggest (i.e., Mulder, 2012). People are obviously more likely to recommend VET in some countries than in others, but not necessarily in countries with high VET participation. Empirical data suggests that there is a link between information on VET and participation rates in VET (CEDEFOP, 2017, 2018). Labor market opportunities are also an explanatory factor for differences in educational and occupational choices (Mulder, 2012). In a difficult employment context, it is safer for students to engage in a more general and polyvalent educational track than in a precise vocational training. While a safety net effect of VET is observed in many countries (OECD, 2017), it appears that the type of VET system plays a role, and in particular, that dual system countries seem to offer more measurable protection to VET graduates (Shavit and Müller, 2000; CEDEFOP, 2018).

Our integrated model builds on two major explanatory strands: on one hand, the contextual and sociological factors, and on the other, the psychological processes within the individual. Both taken together allow to model the individual students’ decisions in relation to their educational and vocational choice.

Regarding the sociological aspects, we have presented a series of theories that help to understand how differences in VET have originated throughout Europe. The works of Bercusson et al. (1992) and Greinert (2005) show how and why different models of VET have emerged, and that the conditions of access and the training offered can vary greatly from one country to another. Maurice et al. (1986) show that VET qualifications can have a different function according to national context. From one country to another, the choice of VET has therefore different consequences. Generally speaking, educational and vocational choices are a major aspect in the reproduction of social inequalities (Bourdieu and Passeron, 1964, 1970; Boudon, 1973; Bowles and Gintis, 1976; CNESCO, 2016; OECD, 2019). VET disproportionately attracts students from low-income backgrounds. This choice is strongly influenced by the students’ families, especially their parents. Parents often think in terms of social class maintenance, which means for children from low-income backgrounds that their parents often feel comfortable with an educational situation where they can reach at most the same socio-economic level. In addition to a preference for the same educational pathways that they have followed themselves, it is in a logic of economic constraint that their parents will tend to prefer short educational pathways that lead quickly to working life. More demanding educational pathways are sometimes avoided because of an overestimation of the probability of failure (Di Stasio, 2017).

The psychological part of our model builds on theories of mental development and mental imagery in relation to vocational choice. When young people are pushed to choose an educational or professional path (Klineberg, 1967) and to build a professional identity (Fusco et al., 2019), this task is influenced by their relationship with adults, and mainly their parents (McGee, 1984; Aronowitz, 2005; Kerpelman et al., 2008). The influence of peers and the social representations surrounding the topic also play a role in the development of a professional identity (Skorikov and Vondracek, 2007; Liechti, 2012). The mechanism of constructing a vocational project is described as being based on mental images, and in particular on the “profession image”. According to Brillet and Gavoille (2016), it is the comparison of profession images with the student’s self-image that guides the educational and vocational choices. One of the first and most important steps in this process is the choice between a vocational or a general educational track. This first choice, which has to be made quite early in most countries can, depending on context, drastically limit the student’s further choices and possibilities as it leads to two different “families” of jobs. The over-representation of students with a modest background in VET tracks (Di Stasio, 2017) suggests that, all other influences set apart, the matching process of profession images and self-image leads them to the VET track. This would imply that their self-image is more in tune with the VET track than with a more general track that can lead to higher studies. Although some students could aim at something different, they may not even envision it.

**FIGURE 6:**
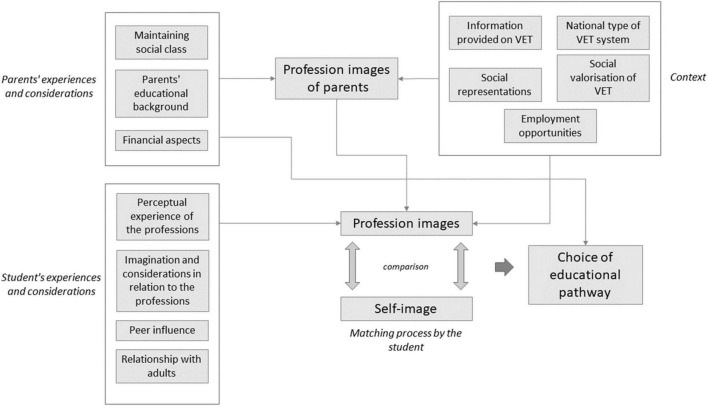
Integrated model of the educational and vocational choice.

Taking into account the above elements, we propose an explanatory model of educational and vocational choice based on three dimensions: the experiences and considerations of the student, those of his or her parents, and the wider context in which the student evolves (see Figure 6).

In this model, the student’s educational and vocational choice is seen as being mainly the result of a comparison that the student makes between his or her profession images and his or her own self-image, with the choice being the result of the best match. Students’ profession images are influenced by their experiences and considerations, i.e., their actual experience of occupations, their (partly fantasized) imagination of professions, the influence of peers and their relationship with adults. At the same time, the students’ parents have their own profession images via their school career, a thinking pattern based on social class maintenance and financial constraints. These parental profession images influence those of the student. Furthermore, irrespective of the result of the matching carried out by the student, and therefore of his or her own preferences, parents can directly influence the choice of educational pathway by imposing their own thinking and their logical assumptions. Finally, the student’s and parents’ profession images are affected by contextual elements such as the national type of VET system, the social representations conveyed, the social valorization (public image) of VET and the information provided about VET.

## Conclusion

Understanding the mechanisms of educational choices allows us to better evaluate the challenges the various national VET tracks in Europe are facing. At the level of the individual student, when making a decision about his or her educational and vocational pathway, we distinguish three dimensions of decision influencing elements: the young person’s experiences and thoughts on professions, the influence of parents, and contextual elements. Vocational choice is therefore a multidimensional decision. A better understanding of VET participation’s mechanisms, and in particular the distinction between individual and societal aspects is an asset in managing and optimizing student guidance. The presented model structure can contribute to empirically measure and analyze the mechanisms of educational and vocational choice.

In the field of VET, several courses of action can be imagined. (1) Action on the public image of VET. Understanding the reasons why the image and social valorization of VET differs greatly from one country to another, and finding ways to improve this image, are avenues to be explored. (2) Awareness of the social background. Ideally, social status should not be a selection criterion for VET. The vocational choice should be made on the basis of students’ interests and abilities. If VET is a second- or even third-class educational pathway in a given school system (where the choice is too often made on negative criteria rather than positive), it is not surprising that its public image suffers. VET should be able to attract young people from all social classes, as it can offer real career prospects and covers a very wide range of occupations to meet the diversity of individual students’ genuine career interests. (3) Action on the “profession image.” As we have seen, young people’s images of different professions and career paths can be as much the product of their imagination and preconceptions rather than of real experience in a professional environment. Vocational choice based on a speculative and erroneous conception of a professional activity can be damaging for motivation regarding training, and also subsequently, in the exercise of that activity. Hence the paramount importance of enabling young students to construct correct, realistic images of existing occupations. It seems that, despite the guidance that is offered, this aspect is still frequently neglected.

In countries or regions where the public image of VET may be unfavorable, a first concrete step would be to conduct a detailed analysis of this situation by carrying out studies on the public image of VET and of the concerned occupations. This can bring a better understanding of why there are discrepancies between different sectors of activity. Such studies may reveal organizational problems requiring a policy response. They may also show that the image of VET and of certain occupations is more negative than the reality faced by students and graduates of these schemes, either due to prejudiced or no-longer relevant preconceptions. Market realities and practical, technical realities of certain trades can change rapidly, often with these changes not being perceived by the general public. A high quality, regularly updated information system on VET can help to build realistic perceptions.

The impact of social background on educational pathways is a classical problem of social reproduction. It involves strong mechanisms related to the functioning of societies, and touches on sometimes strongly held social values. This is why it is not easy to bring about rapid change in this area. A first challenge is to make decision-makers and actors in the field aware of the need to take a bit of a distance from the educational and cultural context with which they are most familiar. We can assume that educational and professional choices are still too often made for reasons related to misunderstandings and misconceptions. Instead of reflecting the skills and interests of students, these choices are too often related to a reflection of existing social structures. In concrete terms, the orientation of students with a view to their educational and professional choices should involve the students’ parents. Information is a key element that should be provided to students and their parents with appropriate emphasis. The cultural and economic differences that result in some countries perceiving VET as a second-choice educational pathway (while in other countries it has a neutral image compared to other educational pathways), is an important aspect to consider.

Finally, in an effort to ensure that young people can create realistic mental images of occupational choices and economic activities, close cooperation between (vocational) schools and companies is an approach to prefer, allowing young people to visit workplaces as much as possible and thus gain a more realistic idea of the nature of working life in a given profession. The building of a realistic profession image features not only having an understanding of the daily practice of that profession, but also being aware of details such as working hours, working conditions, pay and job prospects. Early and multiple contacts with the world of work therefore enables students to make better-informed decisions. Differences in exposure to working life, and access to information about careers may depend on young people’s background. In this respect, it is the responsibility of schools and career guidance services to put students as much as possible on the same level.

## Data Availability Statement

Publicly available datasets were analyzed in this study. This data can be found here: https://appsso.eurostat.ec.europa.eu/nui/show.do?dataset=educ_uoe_enrs04.

## Author Contributions

All authors listed have made a substantial, direct, and intellectual contribution to the work, and approved it for publication.

## Conflict of Interest

The authors declare that the research was conducted in the absence of any commercial or financial relationships that could be construed as a potential conflict of interest.

## Publisher’s Note

All claims expressed in this article are solely those of the authors and do not necessarily represent those of their affiliated organizations, or those of the publisher, the editors and the reviewers. Any product that may be evaluated in this article, or claim that may be made by its manufacturer, is not guaranteed or endorsed by the publisher.

## Footnotes

1 The other two answer options are ‘it depends on the person’ and ‘I don’t know.’

2 “Program for International Student Assessment”. Studies regularly conducted by the OECD.
